# The General Medical Council

**Published:** 1895-06-15

**Authors:** 


### The General Medical Council.

The fifty-eighth session of the General Medical Council of Education and Registration was opened on May 28th, under the presidency of Sir Richard Quain, and terminated on June 6th. Dr. Thorne-Thorne, C.B., F.R.S., was introduced, and took his seat as a Crown representative for England, in the room of Sir John Simon, retired.

The President delivered an address dealing with the past work of the Council, and with the questions likely to be brought forward during the session. For this he received the thanks of the Council, and the address was ordered to be printed in the minutes. Certain customary returns were received from the medical departments of the navy, the army, and the Indian service, and also a letter from the Lord President of the Privy Council, forwarding a copy of a Bill for the Registration of Midwives, which had been introduced into the House of Lords by Lord Balfour of Burleigh, and read a second time, and on which the Lord President desired to receive the opinion of the Council. The Bill was referred to a committee, with instructions to report during the session; the Pharmacopœia Committee was elected; Mr. Bilton Pollard, F.R.C.S., on the nomination of the Society of Apothecaries, was appointed an Assistant Examiner in Surgery to the Society for a term of five years; other committees were appointed; and the Council then adjourned, so as to leave the rest of the day available for Committee meetings.

On May 29th, the first business was to receive a deputation of thirteen gentlemen, professing to be sent by the Lancashire and Cheshire Branch of the British Medical Association, who addressed the Council in opposition to the Midwives Registration Bill. The Council received at the same time a written communication, signed by a large number of members of the branch, who protested against the views which the deputation was expected to advance, and also against the validity of their commission to represent the branch upon the question. Dr. Colin Campbell, Dr. Helme, and Dr. Woodcock addressed the Council, and the deputation then withdrew.

It was announced by the President that negotiations had been concluded for purchasing the freehold of the premises, 299, Oxford Street, occupied by the Council, together with that of the adjoining house, 16, Hanover Square; and that plans had been prepared for the alteration of the offices of the Council in a manner to afford greater convenience for the transaction of business. The action of the President and of the Executive Committee in this matter was approved, and the trustees of the Council were directed to complete the purchase.

The 30th and 31st of May were occupied in the investigation of charges brought by the Medical Defence Union against Mr. Robert Fitzroy Benham, M.R.C.S., who was accused of "infamous conduct in a professional respect" in relation to the Queen's Jubilee Hospital, carried on and conducted by Mr. Benham, and represented by him to be a *bonâ fide* charitable institution. It was alleged:—

(*a*) That the hospital was in no sense a *bonâ fide* charitable institution, but that it was and is a private professional and commercial speculation of Mr. Benham's own.

(*b*) That he had knowingly, and for the purpose of deceiving the public, published inaccurate and misleading accounts of the hospital, and inaccurate and misleading statements as to its conduct and management.

(*c*) That the hospital banking account had been used by him for his own purposes, the hospital moneys and his own moneys being mixed in such account.

(*d*) That the hospital accounts had been manipulated by him or with his knowledge, so as to make it appear as if he personally had advanced and afterwards given to the hospital a sum amounting to £3,200, whereas no such sum was so advanced or given by him.

(*e*) That there was and is no duly qualified resident medical officer or house surgeon attached to the said hospital, and that cases requiring the attendance of a qualified practitioner had been attended by a person or persons who was or were unqualified, and that such unqualified persons were covered in their attendances and practice by him or practitioners on the medical staff of the said hospital at his instance, and that the patients who were treated at the said hospital were thereby deceived and defrauded.

It appeared from the evidence, and was not denied, that the Jubilee Hospital was founded by Mr. Benham, that he became responsible for the expenses incurred in connection with it, and that, regarded from a financial point of view, the conduct of its affairs had been unbusiness-like and ill-advised. His private moneys had become more or less muddled up with those of the hospital, and the result was difficult to disentangle; but there seemed to be no doubt that he had really paid considerable sums to keep the institution afloat, and there was nothing to show that he had derived any pecuniary benefit from it. The length of the inquiry was greatly due to the circumstance that Mr. Benham was defended by three counsel and a solicitor, and that the examinations and cross-examinations of witnesses were very protracted. It is not professionally infamous to be unbusiness-like, and the Council, at the conclusion of the hearing, speedily decided that no evidence to sustain the charges had been put before them. The prosecution seems to have been suggested by some articles condemnatory of the hospital which had appeared in *Truth*, and the editor of that journal, Mr. Horace Voules, was called as a witness in support of the accusation.

On June 1st, Mr. John Crump Lindop was charged with infamous conduct in having "covered" an unqualified person named Wadlow, living at Peterborough; but it was known that Mr. Lindop had left Peterborough since the commencement of the proceedings, and it was not certain that the summons of the Council had reached him. The case was therefore adjourned to the November session.

Mr. George Francis McCarthy was charged with infamous conduct in having "covered" an unqualified person named Coppin, the complaint being made by Mr. Braxton Hicks, in consequence of circumstances which came to his knowledge in the course of his duties as coroner. The Council found that Mr. McCarthy had committed the offence charged against him, but adjourned the further consideration of the case until November, in order to give him an opportunity of proving that he had discontinued the course complained of, and that he had conducted himself in a proper manner during the interval.

Mr. Samuel Frederick Murphy was charged with infamous conduct in having "covered" an unqualified practitioner and ex-convict named Millerchip. He did not attend, but sent a letter of apology through a solicitor. His case was adjourned until June 3rd, and a notice was sent to him to say that his attendance could not be dispensed with. On the 3rd he again failed to appear, and the Council found the charge proved, and directed his name to be removed from the Medical Register. A similar charge was brought against Dr. Charles Farrar by the Medical Defence Union, and was sustained by numerous affidavits. There was also a defence, mainly founded upon affidavits by the same persons, denying the statements to which they had sworn; and, in these circumstances, Dr. Bateman (the Secretary of the Union) withdrew the charges until the whole matter could be fully investigated, and the Council declined to proceed with the hearing.

The name of a person named John Eustace Dennan, who had been convicted of obtaining money by false pretences and sentenced to ten years' penal servitude, was ordered to be erased from the Dentists' Register.

A communication had been received from the Lord President of the Privy Council containing an application from the Netherlands for the admission of Dutch medical graduates or licentiates to the British Medical Register; but, as it appeared from the accompanying documents that British registered practitioners were called upon to pass a final examination before a Dutch Board before being permitted to practise in the Netherlands, the Council resolved to inform the Lord President that the privileges accorded by that country were not such as were contemplated in section 17 (1) of the Medical Act of 1886, and that it was not at present expedient to recognise that the Netherlands should be recognised by Her Majesty's Government as a foreign country to which the provisions of this section should be applied.

The next business, which consumed the greater part of three days, was the consideration of the report of the committee on the Midwives Registration Bill. The committee had not dealt with the principle of the Bill, deeming that this principle had received the approval of the Council in the course of many discussions of the subject during the last twenty years, but went over the Bill clause by clause, in order to suggest such alterations of detail as might remove objections, and might render it more certain to attain the end proposed. As soon as the report of the committee was brought up, however, it became manifest that a small section of the Council was opposed to the principle of the Bill, and wished to destroy all chance of its becoming law. This section was led by Sir Walter Foster and Mr. Wheelhouse, the direct representatives of the profession for England, and included also Dr. McVail, of Glasgow, whose concern in the matter, as this Bill is not framed to apply to Scotland or Ireland, was not at first sight very manifest. In former years, Sir Walter Foster had energetically supported in the Council the kind of legislation which he now opposed; and, in 1889, he moved the following resolution, which was seconded by Dr. Glover, and agreed to:—

"That this Council regards the absence of public provision for the education and supervision of midwives as productive of a large amount of grave suffering and fatal disease among the poorer classes, and urges upon the Government the importance of passing into law some measure for the education and registration of midwives."

In total disregard of this resolution, and of others of the same kind which he had moved or supported on former occasions, Sir Walter now made himself the spokesman of an opposition to the Bill which has been organised by a small number of medical practitioners in the North-East of England, who appear to be afraid of the competition of an improved class of midwives in their practice. It was said that both he and Mr. Wheelhouse received one morning a telegram form the prime mover in the organisation referred to, and that they both decided to abandon their former convictions, and to speak and vote against the Bill. Dr. Glover, the remaining direct representative for England, stood manfully to his guns, and, after a succession of divisions in which the opponents of the Bill were beaten by large majorities, the report of the committee was in substance adopted, and the approval of the Council was given to the Bill in an emphatic manner. It is none the less to be feared that Sir Walter Foster's opposition may be more effectual with the Government than it was with the Council, and that there will be little disposition, in the present state of public affairs, to afford active support to a measure which is distasteful to the Parliamentary Secretary of the Local Government Board.

The only remaining business of importance was the presentation by the Examinations Committee of the Council of two reports, the first on the final or qualifying examination of the Conjoint Board formed in Dublin by the Royal College of Surgeons of Ireland and the Irish Apothecaries' Hall, the second on the final examinations of all the qualifying bodies. The Council's visitor and inspector, last year, reported so unfavourably of the Irish conjoint examination referred to that the Council ordered it to be inspected again on every occasion of its being held; and now decided, upon the consideration of four reports, to represent to the Privy Council that the examination, as far as the department of medicine is concerned, is "insufficient" to satisfy the requirements of the Medical Act and to afford proper protection to the public. In the meanwhile, the Irish College of Surgeons had already decided to break the combination, and had given notice to that effect, so that no more examinations of the kind will be held, and the Privy Council will probably not feel called upon to interfere. In that case, it will be open to the Irish Apothecaries' Hall to apply to the Medical Council to appoint to it examiners in surgery, and it will have a right of appeal to the Privy Council if this application should not be acceded to. On the whole, there seems to be some probability that the Irish Hall will cease to act as a licensing body, and, in any case, it will not be in a position to hold its customary summer examination.

The second report, on the final examinations generally, is mainly of a satisfactory character, but it is followed by suggestions for several amendments in matters of detail, and, on account of the length to which the session had been protracted, it was resolved to defer the consideration of these suggestions until November, when, if approved, they will be issued as "recommendations" by the Council.

The report of the Pharmacopœia Committee was received and adopted, and a sum of £3,000 towards the expense of preparing a new edition of the work was placed at its disposal. This new edition is intended to be "Imperial" instead of "British," and to supply the requirements of India and the Colonies, as well as those of the mother country.

